# Very Slow Search and Reach: Failure to Maximize Expected Gain in an Eye-Hand Coordination Task

**DOI:** 10.1371/journal.pcbi.1002718

**Published:** 2012-10-11

**Authors:** Hang Zhang, Camille Morvan, Louis-Alexandre Etezad-Heydari, Laurence T. Maloney

**Affiliations:** 1 Department of Psychology, New York University, New York, New York, United States of America; 2 Center for Neural Science, New York University, New York, New York, United States of America; 3 Department of Psychology, Harvard University, Cambridge, Massachusetts, United States of America; Northwestern University, United States of America

## Abstract

We examined an eye-hand coordination task where optimal visual search and hand movement strategies were inter-related. Observers were asked to find and touch a target among five distractors on a touch screen. Their reward for touching the target was reduced by an amount proportional to how long they took to locate and reach to it. Coordinating the eye and the hand appropriately would markedly reduce the search-reach time. Using statistical decision theory we derived the sequence of interrelated eye and hand movements that would maximize expected gain and we predicted how hand movements should change as the eye gathered further information about target location. We recorded human observers' eye movements and hand movements and compared them with the optimal strategy that would have maximized expected gain. We found that most observers failed to adopt the optimal search-reach strategy. We analyze and describe the strategies they did adopt.

## Author Summary

A variety of human daily activities, such as cooking, drawing, and driving, involve coordination of eye and hand. Typically your hand moves towards whatever you have just looked at. But is this coupling compulsory? To test whether human observers can adopt appropriate eye-hand coordination strategies to maximize rewards, we created an unusual task where good performance required that hand and eye move independently of each other in order to rapidly find and touch a target. Observers were rewarded for minimizing the overall time to find and touch a target among distractors and we made the visual search and hand movements very slow so that a simple “hand-follows-eye” strategy would reduce observers' winnings considerably. Most observers failed to choose the optimal visual search strategy but did intelligently coordinate hand movements to the visual search strategy they did pick. The “very slow search and reach” task we developed provides a novel approach to investigate coordination between perceptual and motor systems experimentally and computationally.

## Introduction

In visually guided manual tasks that involve a sequence of targets, the movements of eye and hand are usually tightly linked [1]–[4]. For example, in making a peanut butter and jelly sandwich at home, you would typically fixate the jar of peanut butter while you move your hand toward it [5]. In such a task the relationship between eye and hand is simple: The hand always waits for the eye to fixate the next target and then “follows the eye”.

This strategy of coordination makes sense, intuitively. Shortly after the start of sandwich making, you know where all the relevant items are and, if you did not use your gaze to aid your reach, it is unclear what you might do with your eyes instead. If, however, there were a rewarding alternative (e.g. watching your favorite television show), we might expect very different eye and hand movements in carrying out the same task.

When we talk about rewards and the probabilities of rewards, we are in the framework of statistical decision theory [6], [7]. The eye and the hand have potentially infinite ways to coordinate with each other. Statistical decision theory allows us to predict the eye-hand strategy that maximizes expected gain and compare human performance to ideal.

In the past decade several groups of researchers have compared human choice of hand or eye movements to the performance of ideal decision makers who plan movements to maximize expected gain or a similar criterion [See 8 for a review]. Researchers evaluating eye movement selection in visual search [9]–[11] or reading [12], [13] have reported that human performance is close to optimal. Researchers evaluating reaching movements have found similar, near optimal, performance in spatial [14], [15] or temporal [16], [17] reaching tasks. In this article we examine human performance in a task where participants plan a series of inter-related eye and hand movements, searching for a target and reaching to touch it. We compare human performance to ideal performance maximizing expected gain.

**Figure 1:**
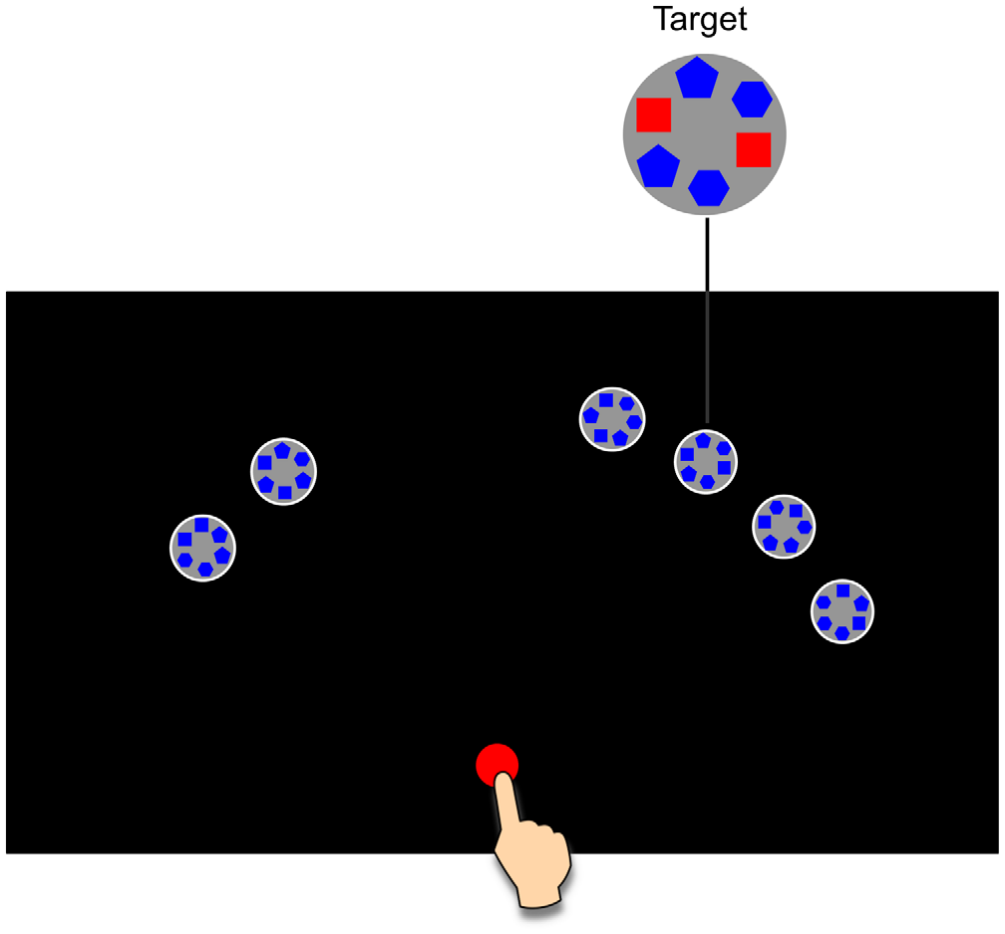
An example of the stimulus array. The red circle is the starting position for the eye and the hand. Each gray circle with blue shapes inside is an object. Two clusters of objects are located to the left and right of the midline, on a virtual arc centered at the starting position. One cluster contains four objects, the other two. On half the trials the two-cluster is on the left as shown, on the other half, on the right. Each object is equally likely to be the target. One and only one of them is the target to be touched. See the Stimuli section for a definition of the target and distractors.

We investigated an eye-hand coordination task where we created an unusual reward structure intended to encourage a decoupling of the eye and the hand. Human observers were asked to find and touch a target among five distractors (Figure 1). The monetary reward they received for touching the target decreased linearly with the total search-reach time, the time from the appearance of the search arrays to touching the target. We were interested in how much observers could reduce the search-reach time by coordinating eye and hand appropriately. While there is evidence that the mode of eye-hand coordination may vary with experience [18] or skill [19], it is unknown whether people would choose eye-hand coordination patterns that would maximize expected gain.

In the task, we intentionally slowed down both visual search and hand movements to amplify their temporal costs. This sort of constraint occurs in everyday movements when, for example, we carry a very full cup of tea from one place to another. Visual objects (target or distractor) were made so complex that observers had to fixate an object for 1∼2 seconds to discriminate target from distractor. Observers were required to move their finger along the surface of the touch screen under a speed limit. It took about 9 seconds to cover the distance from the starting position to any of the objects. As mentioned above, the observer received a monetary reward for touching the target, a reward that decreased linearly with time since the beginning of the trial.

We designed that task so that a sequential strategy that consisted of first locating the target and only then initiating the finger movement away from the starting position to the target would result in negligible reward. Participants could do considerably better by starting their hand movement before they had located the target through visual search. The strategy maximizing expected gain required that they plan hand movements on incomplete information about the target and update their movement plan as further information about the target location became available through visual search. If, for example, visual search of all the targets on the left half of the display screen has failed to find the target, then the target must be on the right side and the trajectory of the hand can be adjusted to take advantage of this additional knowledge.

The optimal strategies for an individual depend on the individual's speed in searching for the target, her movement speed, and the spatial layout of the target and distractors. As Figure 1 illustrates, the to-be-searched objects were spatially divided into two clusters, one containing two, and the other four objects, located to left and right of the midline of the display. Each object was equally likely to be the target.

We developed a model of optimal eye-hand coordination described under the Methods section. Intuitively, the optimal initial movement strategy is to move more towards the larger cluster than the smaller cluster, for the former has a larger probability (4 out of 6) of containing the target. However, the optimal eye movement search strategy is to first search the *smaller* cluster. After only two searches, the observer will know whether the target is on the side with only two tokens or on the unsearched side with four tokens. This knowledge quickly reduces the spatial range of the possible aims of the reach movement.

We recorded observers' eye movements and hand movements. Before the search-reach task, observers were trained in visual search with key press responses and in moving on the touch screen, and during these training sessions we obtained their search slope and reach speed separately. We compared the performance of human observers to the performance predicted by our model of optimal eye-hand coordination (maximizing expected gain) described below.

We considered three questions. First, do people use the visual search strategy that maximizes expected gain? In particular, do observers search in the order that reduces the spatial uncertainty of the target most quickly? We will conclude that they do not.

Second, the uncertainty of the target changes as the visual search proceeds. At the beginning of a trial, any of the six objects could be the target. When certain objects have been identified as distractors, though the target is still unknown, the target can only be one of the remaining objects. Can we find evidence that observers adapt their hand movements to the partial information acquired through visual search before the target location is identified? In particular, do they move their hands at all before the target location is known?

Finally, if people failed to maximize expected gain in visual search and/or hand movement, could this failure be attributed to a hard constraint of the motor system? Is it possible, for example, that the hand has no choice but to follow the eye, whether appropriate or not?

## Methods

### Ethics statement

The experiment had been approved by the University Committee on Activities Involving Human Subjects (UCAIHS) of New York University and informed consent was given by the observer prior to the experiment.

### Apparatus

Stimuli were presented in a dimly lit room on a 32-in. (69.8×39.2 cm) Elo touch screen, which was vertically mounted in a Unistrut frame and was run at a frame rate of 60 Hz with 1366×768 resolution in pixels. An Eyelink II eye tracker was used to record the gaze positions of the observer. The display and recording were controlled by a Dell Pentium D Optiplex 745 computer using the Psychophysics Toolbox [20], [21] and the Eyelink Toolbox [22]. A chinrest was used to help the observer to maintain a viewing distance of 50 cm, at which distance 1 cm at the center of the screen approximately subtended 1.1 deg. The observer wore a single finger cut from a cloth glove to reduce the resistance of movement. A touch screen calibration procedure was performed for each observer before the experiment.

### Stimuli

An example of the stimuli in the search-reach task is shown in Figure 1. Each object (target or distractor) was a white-lined gray circle (2 cm in radius), within which six shapes were evenly distributed. Every two shapes opposite to each other formed a pair. The shapes were equal in area and could be square, pentagonal, or hexagonal. If all of the three pairs of an object were made of two different shapes, the object was a distractor; the target had exactly one pair that consisted of the same shapes. In Figure 1, the target was the third object from the right.

The starting position for the finger was a red circle of 0.8 cm radius, which was on the midline of the display and close to the bottom. Two clusters of objects were located to left and right of the midline, one cluster containing four objects, the other two. All the objects were along a virtual arc centered at the starting position. The center-to-center distance from the starting position to any object was 27 cm. Within a cluster the objects were equally spaced and the space between any two adjacent objects was 3 cm. That is, with the starting position as the origin, centers of two adjacent objects spanned an arc of 15 deg. The centers of the two clusters were 45 deg left and 45 deg right of the midline. On each trial the objects as a whole might have a clockwise or counter-clockwise jitter of no more than 3.8 deg.

### Procedure and design

The experiment consisted of two 1.5-hour sessions on two different days. All observers went through the following three experimental phases corresponding to three different tasks: training of visual search, training of reach, and testing of search-reach. In each trial of a particular task, observers receive bonus points for successfully performing the task. During the trial, the number of points that could be won started at 100 and decayed linearly with the time used (at a rate of 8, 7, and 5 point/sec, respectively, for the three tasks) until the task was successfully completed or the count reached 0. The observer received the point count remaining at the end of each trial. Every 1000 points were redeemed as US$1 at the end of the experiment.

#### Training of visual search

The task in this phase was ordinary visual search – to search for a target among distractors. A trial began with the display of a red circular starting position on the touch screen. When observers put their finger on the starting position and fixated it for 0.5 second, six objects appeared, in clusters of two and four. In half of the trials, the small cluster was on the left and the large cluster on the right; in the other half, the reverse. Observers knew that the target was equally likely to be any of the objects. They kept their finger on the starting position during the visual search. When they found the target, they responded by lifting their finger while fixating the target. Feedback followed. If they had correctly indicated the target, they were informed how many reward points they had won in the trial. Otherwise they were informed that they had erred and would receive no reward.

Each observer completed three variants of the task. In the *small-cluster first* task, observers were required to search the small cluster first. If they fixated any object in the large cluster first or returned to an object in the small cluster after they had visited the small cluster, the trial would be cancelled and they received a warning message. In the *large-cluster first* task, in contrast, observers were required to search the large cluster first. By including the small-cluster first and large-cluster first tasks, we induced observers to explore different orders of search. The order of these two tasks was counterbalanced across observers. Afterwards, in the last part of visual search training, the *free search* task, observers were left free to choose the order of search.

The layout of the objects could be small cluster on the left and large cluster on the right or the reverse. The observer performed 12 trials in the practice and 6 (target location)×2 (layout)×8 = 96 trials in the formal experiment for each of these three tasks. These tasks provided observers with experience at the visual search task. At the same time, we could estimate the search slope (searches/second) as well as the preference in search order for each observer in the free search task. During visual search training no hand movements were involved except to initiate the trial by touching the red dot and terminate it by releasing the red dot.

#### Training of reach

Observers fixated and pressed the starting position to start the trial, just as they did in the visual search tasks above. At the locations of the to-be-searched objects, one white circle and five blue circles appeared instead. The task was to move one's finger along the surface of the screen into the white target and then lifted the finger. Observers were required to move at a speed of no more than 4 cm/s. The number of reward points for successfully reaching the target linearly decreased with the movement time. The feedback was similar to that of the search tasks.

There were 12 practice trials and 6 (target location)×2 (layout)×8 = 96 experimental trials. In practice trials, a white circle (radius 1 cm) followed the movement of the subject's finger on the screen with the subject in effect “dragging” the circle from point to point on the screen. The speed of the circle was limited to 4 cm/s and the positions of the finger and the circle were recorded every 16.7 milliseconds. If the finger moved too rapidly and opened a gap between the center of the circle and the finger of more than 1 cm, the trial would be terminated with a warning message displayed. Thus, over the course of training, the subject learned to move smoothly on the screen without “losing” the circle. We applied the same speed limit algorithm during the main part of the experiment but the circle was not visible. A trial was cancelled and repeated later if the requirements of the movement task were violated.

Parallel to the training of visual search, the goal of the training of reach was two-fold. On one hand, it helped observers to move comfortably on the screen under the speed constraint. On the other hand, it enabled us to measure the actual speed for each observer and detect possible motor biases.

#### Test of search-reach

After the separate tasks of visual search and reach, in this phase, observers were tested with the search-reach task as described in the Introduction. The search-reach task was the same as the free search task except that observers indicated the location of the target by moving their finger to it. They followed the same speed constraint as in the training of reach. The rewarding points for successfully finding and reaching the target were based on the time from the appearance of objects to the touching of the target. Feedback was similar to that given in the search and reach tasks.

Each observer performed 12 practice trials and 6 (target location)×2 (layout)×8 = 96 experimental trials for the search-reach task. We did not repeat unsuccessful trials in the test of search-reach. If observers failed on a trial, they lost the bonus for the trial.

Observers completed the training of visual search in the first session and the training of reach and test of search-reach in the second session. For all the phases, the gaze positions and the screen coordinates of the finger were recorded every 0.017 second.

### Observers

Eight observers (four female) participated. None were aware of the purpose of the experiment or the hypotheses under test. All were right-handed and used their right index finger to move on the touch screen. They received US$12 per hour plus a performance-related bonus calculated as described above.

### Model of optimal eye-hand coordination

What concerned us was the strategy of eye-hand coordination people would use in the search-reach task. Based on statistical decision theory [6], [8], [23], we modeled an ideal observer who chooses the visual search strategy and the hand movement strategy that jointly maximize her expected gain. We compared each observer's performance with that of the ideal observer who is endowed with the same visual search and hand movement capacities as the actual observer.

**Figure 2:**
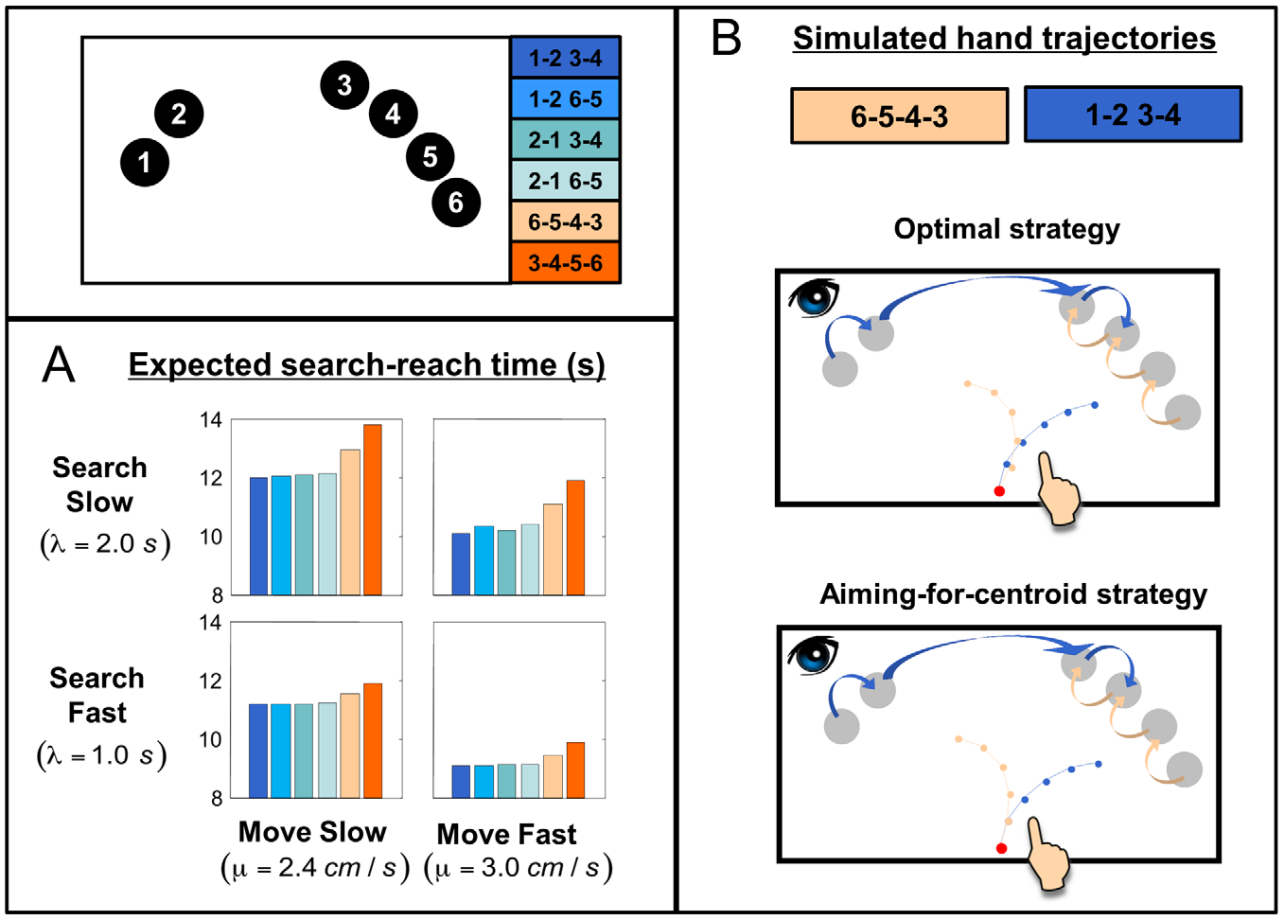
Optimal strategies of eye-hand coordination. **A. Simulated expected search-reach time as a function of the order of search**. Each panel is for a different search and hand movement capacity. The objects are numbered from 1 to 6 (inset). Orders of search are indexed with sequences of numbers. For instance, 12 34 denotes the sequence of fixations: object 1 first, then 2, then 3, then 4, and finally either 5 and 6 or 6 and 5 in either order. See text. Different colors denote different orders of search. We assumed that the observer always uses the optimal strategy of hand movement that minimizes her expected search-reach time under the specific order of search. The displayed ranges of search time per object, 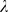, and hand movement speed, 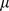, include those for human observers measured in the experiment. Whatever the search or movement speed of the observer, the search order 1234, i.e. starting from the end of the small cluster and moving continuously towards the large cluster, leads to the minimum expected search-reach time. But note that in the range of subjects' visual search speed and hand movement speed, the difference between this optimal order and the other orders are larger when search is slower or hand movement is faster. The difference between 1234 and the other three orders starting from the small cluster (1265, 2134, and 2165) is negligible, but these four are obviously better than the two orders that start from the large cluster (6543 and 3456). For example, for a typical human observer (O7) with 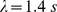 and 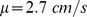, the simulated expected search-reach time of 1234 is 10.3 *s*. The other three orders of small-cluster first cause an increase of less than 0.1 *s*, but the larger-cluster first orders, 6543 and 3456, would cost 0.7 *s* and 1.2 *s* more. Given the reward structure, using the latter two inferior search orders would lead to a loss of 6% and 12%, relative to the optimal one. **B. Simulated optimal and close-to-optimal hand movement strategies**. The hand trajectories are simulated for a typical human observer (Observer 07) with 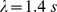 and 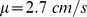. Grey filled circles denote objects. The red filled circle at the bottom denotes the starting position of the finger. We illustrated the trajectories of optimal or close-to-optimal hand movement for two search orders, in different colors, blue for 1234, orange for 6543. Sequences of block arrows on the objects show the search orders. The lines originated from the starting position correspond to the trajectories of the finger. Each dot on a line marks the time when a new object gets identified. The optimal strategy for the hand (top) is the strategy that minimizes the expected search-reach time given a specific search order. The aim-for-centroid strategy (bottom) is to aim for the centroid of the objects that are still unidentified. The differences between the two panels are subtle. If the observer updates her movement aim after the identification of each new object, the expected search-reach time is almost the same as that of the optimal strategy.

We number the six objects as 1∼6 starting from the outmost object of the small cluster (Figure 2, inset). The monetary rewards the observer would receive in the search-reach task are proportional to 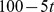, where 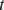 is the search-reach time, i.e. the time from the appearance of the search arrays to the touch of the target. Maximizing expected gain, in the current rewarding structure, thus amounts to minimizing the expected search-reach time, formulized as:
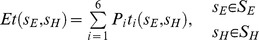
(1)where 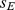 and 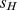 respectively denote the strategies for the eye and the hand, 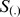 denotes the corresponding set of all possible strategies, 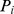 denotes the probability of the *i*-th object to be the target, which equals 1/6 because each of the six objects has the same probability to be the target, 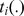 denotes the search-reach time when the *i*-th object is the target.

Both the visual search strategy and the hand movement strategy of interest apply up to the time point when the target is found, since after that the only admissible strategy is to move one's finger straightly towards the target at full speed. The search-reach time is the sum of the time to find the specific target plus this additional reach time. The former is determined by the search strategy alone. The latter is determined by the location of the finger when the target is found, which in turn is determined by the search and hand movement strategies.

To make the optimality problem tractable, we made some reasonable assumptions about the process of search and reach. First, we assumed that the observer fixates and examines one object at a time and does not switch to the next target until the current object is correctly classified as target or non-target. Second, we assumed that it takes a constant time to saccade to a target and then identify it to be the target or a distractor. The actual time will differ with length of saccade, of course, but the differences are negligible at the time scale of the experiment. Third, we assumed that the observer changes the aim of her hand movement only when a new object is identified.

With these assumptions, the visual search strategy is reduced to specifying the order for the eye to visit the objects, such as 123456 or 342516, while the hand movement strategy could be specified by changes in direction of the finger each time a new object is identified. Let 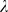 be the time to saccade to and identify one object and 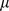 be the speed of hand movement. Denote the location of the *i*-th object as 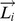. For specific visual search and hand movement strategies, when the *i*-th object is the target, the search-reach time is:
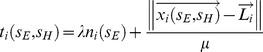
(2)where 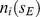 denotes the number of objects visited up to and including the point when the target is found, 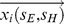 denotes the location of the finger on the screen when the target is found.

Substituting Equation 2 into Equation 1 and cancelling 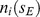, we could express the expected search-reach time as a function of 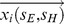, 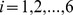. To minimize the expected search-reach time, we used the two steps as stated in the Introduction: First, for any specific search order, we could select the six 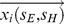 that minimizes the expected search-reach time at the search order. The choice of 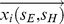 is constrained by the speed of hand movement. Next, we chose the search order that corresponds to the minimum expected search-reach time.

Considering that the last two objects are actually interchangeable in their order, we only need to specify the first four objects to be searched. We simulated all of the possible permutations (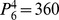) for varying search slope and movement speed and found that the search order of 1234 always led to the shortest search-reach time, assuming that the hand strategy is optimal contingent on the search order. More generally, the search orders starting with the small cluster (1 or 2), on average, had a shorter expected search-reach time than those starting with the large cluster (3, 4, 5, or 6). We called the former the *small-cluster first* strategy, the latter the *large-cluster first* strategy.

For simplicity, we assume that the observer does not switch to the other cluster before finishing one cluster and goes from one end to the other end within each cluster. In Figure 2A, we plot the simulated expected search-reach time for all the six possible search orders: 1234, 1265, 2134, 2165, 6543, 3456. Different panels are for different conditions of search slope 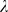 and movement speed 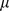.

The differences between 1234 and the other three orders that start from the small cluster (1265, 2134, and 2165) are negligible, while these four are obviously better than the other two orders that start from the large cluster (6543 and 3456). To have an idea of the magnitude of the difference, consider a typical human observer (O7) with 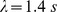 and 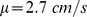. The simulated expected search-reach time of 1234 is 10.3 *s*. The other three orders of small-cluster first cause an increase of less than 0.1 *s*, but the larger-cluster first orders, 6543 and 3456, would cost 0.7 *s* and 1.2 *s* more. Given the reward structure, using the latter two inferior search orders would lead to a loss of 6% and 12%, relative to the optimal one.

We illustrate the simulation of hand strategies for the typical observer (on 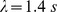 and 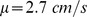) in Figure 2B. The lines originated from the starting position correspond to the trajectories of the finger. Each dot on a line marks the time when a new object gets identified. We illustrate the trajectories for two different search orders, 1234 (in blue) and 6543 (in orange). The three panels are for different hand strategies. The optimal hand strategy (top panel) is the one that minimizes the expected search-reach time under a specific search order, based on the assumption that the observer updates the aim of the movement every time a new object is identified. We obtained it using a nonlinear optimization method (the Nelder-Mead simplex method implemented as “fminsearch” in Matlab).

The computation outlined so far may be too complex for humans to execute. So we considered a heuristic: always move toward the centroid of the objects that have not been identified yet. The observer might update her aim of movement after the identification of each new object (bottom panel), or only after one cluster has been identified, or never unless the target is found. We found that the aim-for-centroid strategy is a good approximation to the optimal strategy. If the movement aim is updated after each object, the expected search-reach time is almost the same as that of the optimal strategy. Even the aim-for-centroid with cluster updating is close to optimal, corresponding to 97%–99% of the maximum expected gain. Even if the aim is never updated, i.e. the hand keeps moving towards its initial direction until the target is found, the expected gain is 91%–96% of the maximum.

## Results

Unless otherwise stated, the significance level used for all tests was .05 with a Bonferroni correction for 8 observers (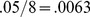).

### Data pre-processing

We used Kumar's [24] algorithms to separate saccades and fixations, with a distance threshold of 0.5 cm for saccades. Given that the radius of an object was 2 cm and the minimum gap between two objects was 3 cm, we defined a circle of 3.5 cm radius around an object as the interest area of the object (approximately 3.9 deg). One fixation or a continuous series of fixations that fell in the interest area of an object and lasted at least 0.3 s in total was taken as a visual examination of the object. For each trial, we obtained the order of search and the timing of each examination.

In the search-reach task, observers failed to touch the target in a considerable percentage of trials. We noticed that the failures of quite a few trials were due to a violation of the speed limit just after the eye fixated the target. We defined these trials as “almost-successful” trials. Most of the speed violations occurred immediately after the target was found and before that there was no significant difference between the almost-successful trials and the real successful trials in movement speed. Across observers the percentage of almost-successful trials ranged from 18% to 30% (median: 21%). The percentage of successful plus almost-successful trials was above 92% for all observers except one observer (O5, 75%). We completed the almost-successful trials by assuming that thereafter the observer would move towards the target at her mean movement speed. The rewards of almost-successful trials were re-calculated based on the re-calculated search-reach time. In later analyses, these completed almost-successful trials were treated as successful trials. Since the violation of speed limit occurred after the target had been identified, there was no reason to assume that observers had used a different search or reach strategy in the almost-successful trials from that which they had used in the real successful trials, although they received no rewards for the former.

### Search slope and movement speed

Through the free search task in the training of visual search, we could estimate how rapidly each observer could identify an object as target or non-target. For each observer, we fitted the search time of a trial as a linear function of the number of objects fixated in the trial. Only successful trials were included. The variance explained ranged from 77% to 96% across observers.

**Figure 3:**
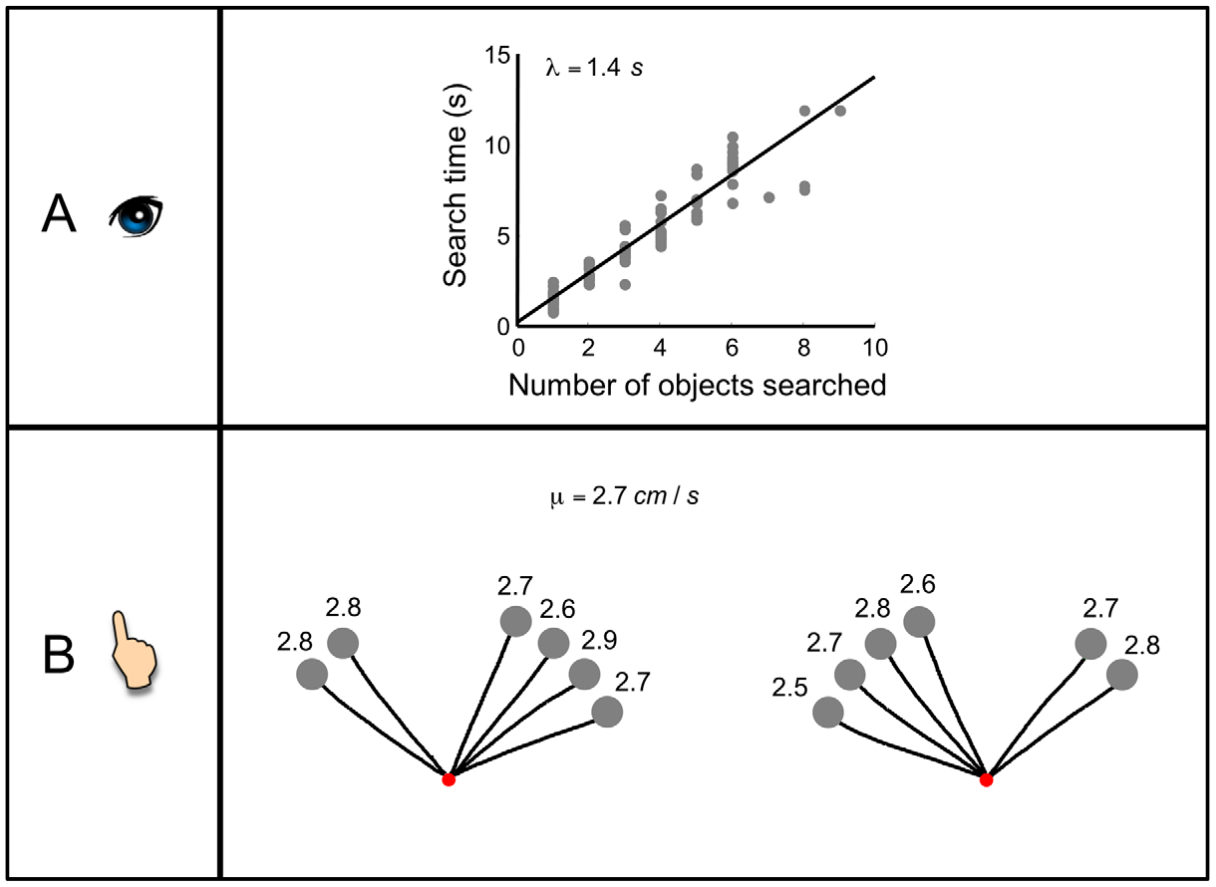
Search slope and movement speed. **A. Search time as a linear function of number of objects searched**. Data of a typical observer, O7, in the free search task. Each grey dot is for one trial. The black line is the fitting line, whose slope corresponds to the time to identify one object, 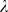. **B. Average trajectories for different target positions**. Data of the same observer O7 in the training of reach. Red filled circle denotes the starting position. Grey filled circles denote positions of objects. In each trail, the observer moved the finger from the starting position to one designated position. Black lines denote trajectories of hand movement averaged across trials. The number above an object denotes the measured movement speed towards the object (*cm/s*). Note that the movement speeds differ little in different movement directions. The mean speed across all target positions is regarded as the observer's movement speed, 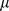.

Figure 3A shows the results for a typical observer (O7). The slope of the fitting line corresponded to the search time per object, 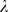. The intercept could be taken as a constant extra time to perform the search task. Across observers, it took 1.2 to 2.0 s per object to classify the object as target or distractor. The extra search time of most observers was close to zero. Only one observer's (O4) extra search time (1.7 s) was significantly greater than zero.

We measured the observer's hand movement speed in the reach task of the training phase, in which the observer moved straightly from the starting position to the designated target position. Figure 3B shows the average trajectories of different target positions for a typical observer (O7). The movement speed was little influenced by the direction of movement. For each observer, we computed the mean speed across all target positions and took it as the observer's movement speed 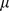. It varied from 2.5 cm/s to 2.9 cm/s across observers. In the text accompanying Figure S2, we discuss why we would expect the observer to move more slowly than the speed limit of 4 cm/s.

### Eye-hand dynamics in the search-reach task

**Figure 4:**
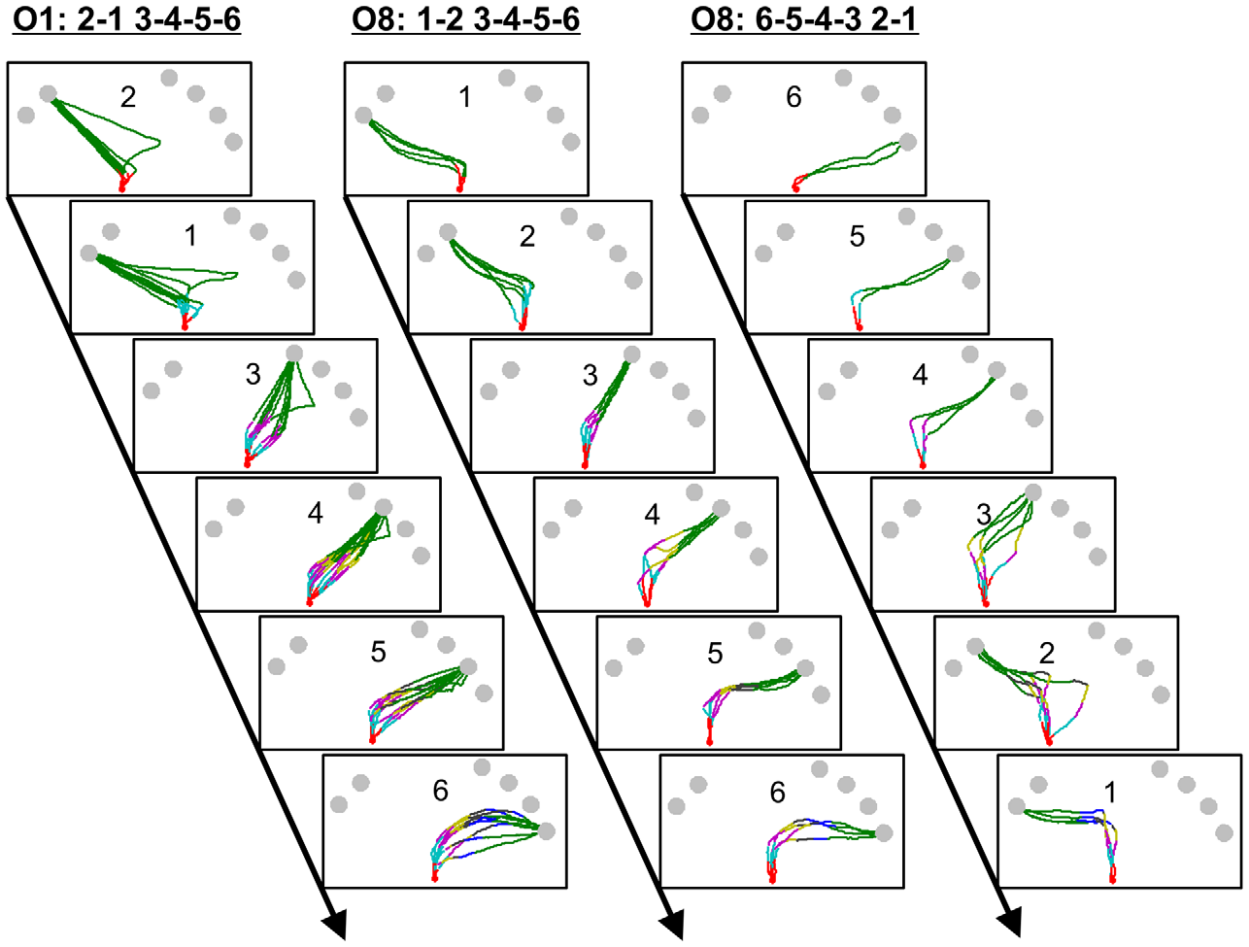
Eye-hand dynamics in the search-reach task. Hand trajectories in the search-reach task are plotted for two typical observers, O1 (left sequence) and O8 (middle and right sequences). Each sequence is for one specific search order (labeled at top). Each panel is for one specific target position (labeled in the panel). Each trajectory is for one trial. Colors along the trajectories code stages of visual search. Red denotes that no objects have been examined. Cyan denotes that 1 object has been examined, and so on. Green denotes that the target has been found. Note where the hand trajectories change going directions and how the trajectories vary with search order.

Figure 4 shows the hand trajectories in the search-reach task for two typical observers and how the movement direction of the hand changed with the updating of visual search (for a demo of one trial, see Video S1). Starting with red, the colors along the trajectories sequentially denote 0, 1, 2, 3, … objects had been identified. Green denotes that the target had been found. Observers O1 and O8 differed in their major search orders: O1 almost always searched in the order of 2134, while O8 used the orders 1234 and 6543 equally often. Concerning the dynamic interaction between the eye and the hand, they had some common features: First, the hand seldom moved straight towards the position where the eye was examining. Rather, the hand tended to move towards future fixation positions. Second, the hand trajectory underwent updating with the identification of target or distractors.

### Efficiency of the search-reach task

Based on the search slope and movement speed of an observer, we could predict her maximal expected gain in the search-reach task with the optimal model described earlier. Efficiency was defined as the average gain of successful trials divided by the maximal expected gain. To avoid overestimating maximal expected gain and thus underestimating efficiency, we added the observer's extra search time, if positive, when computing her minimum expected search-reach time.

**Figure 5:**
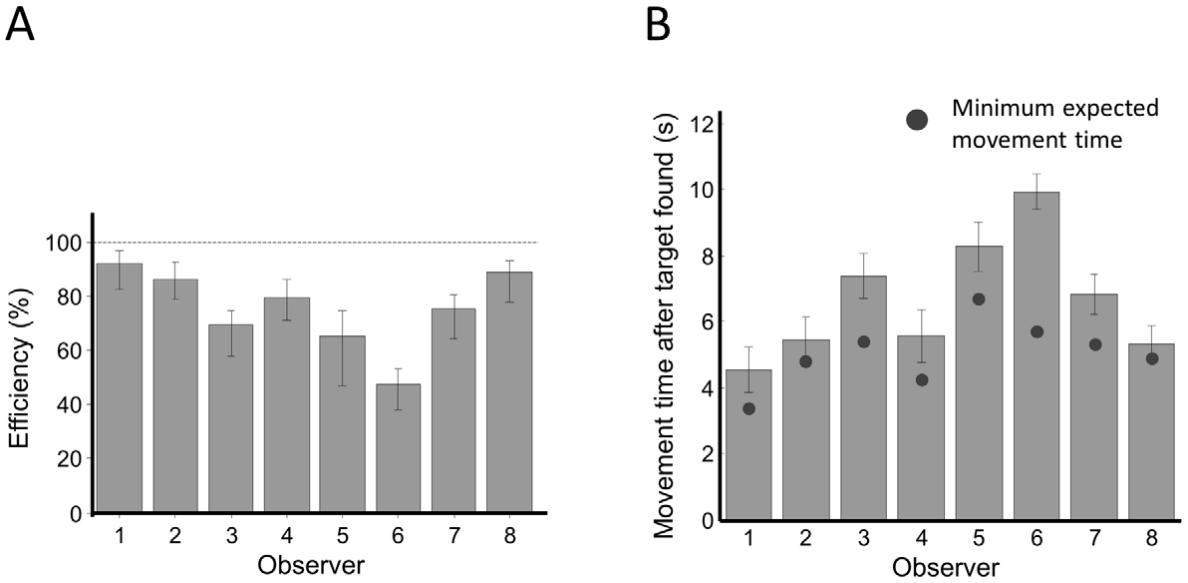
Search-Reach: Human observers' performance compared to optimal. **A. Efficiency**. Efficiency was defined as the average gain of successful trials divided by the maximal expected gain. Most observers were far from optimal. The median efficiency across observers was 78%. **B. Movement time after the target was found**. Black dot denotes the expected movement time after the target was found if the observer used the optimal visual search and hand movement strategies. All the observers' mean post-found movement time were larger than the minimum expected movement time. For 6 of the 8 observers, the difference was significant. In both A and B, each bar is for one observer. Error bar denotes the 95% confidence interval.

We plotted the efficiency of the search-reach task for each observer in Figure 5A. The 95% confidence interval was computed using a bootstrap method [25] with 10,000 resamples.

We could reject the hypothesis of optimality for all observers. Most observers were far from optimal. A median observer achieved only 78% of the expected gain predicted by the optimal strategy of eye-hand coordination. We will look at their actual visual search and hand movement strategies next.

As a second index of task performance, we computed the mean movement time after the target was found (post-found time) for each observer and compared it to the expected post-found time for optimal strategies (Figure 5B and Figure S3). In the search-reach task, the expected time before the target was found did not vary with strategies. The post-found time reflected how close the hand had been to the target at the end of the visual search, that is, how efficiently the hand had used the search time to approach the target. We find the same pattern as we found in considering efficiency: All the observers' mean post-found movement time were larger than what predicted by optimal strategies. For six of the eight observers, the difference was significant.

### Visual search strategy

For the search-reach task, we examined the search order of the trials in which at least one object had been searched. Only 14 trials were missing (O3: 1; O5: 12; O7: 1). As a median observer, the search order of 85% of the trials was one of the six that we had simulated earlier: 1234, 1265, 2134, 2165, 6543, 3456 (see Figure S4A for the percentage of each search order).

**Figure 6:**
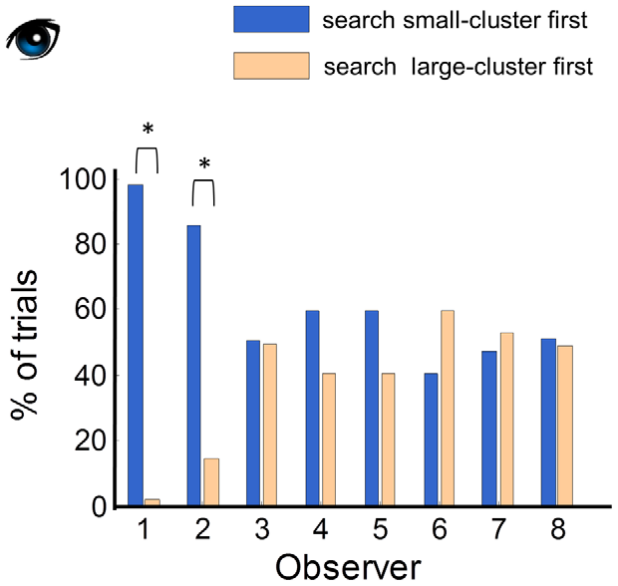
Visual search strategies in the search-reach task. To save time, observers should search the small cluster first rather than the large cluster first. The percentages of usage of the two visual search strategies were contrasted with each other. Star denotes a significant difference. Only two observers correctly used the small-cluster first strategy more often than the large-cluster first strategy. The rest of the observers showed no significant preference between the two.

As we illustrated in Figure 2A, the small cluster first strategies (starting search with 1 or 2) are superior to the large cluster first strategies (starting search with 3, 4, 5, or 6). We tested whether human observers chose to use the small-cluster first strategy to save their search-reach time. The percentages of trials of the two visual search strategies were contrasted in Figure 6. According to a two-tailed binomial test, only two observers (O1 and O2) correctly searched the small cluster first more frequently than the large cluster first. The rest observers showed no preference between the two strategies. Thus, most observers failed to use the optimal visual search strategy.

The two observers who correctly searched the small cluster first in the search-reach task did not do so by accident. In the free search task of the training phase, where the order of search did not influence the cost of time, O1 searched in both orders equally often, and O2 exhibited the reverse preference (large-cluster first). In contrast, observers who happened to prefer the small cluster first in the training phase (O4 and O5) unfortunately gave up this preference in the search-reach task (see Figure S4BC for search preferences in the training of free search).

### Hand movement strategy

When the position of the target is known, there is no doubt the observer should move towards the target at her full speed, as in the reach training task. The interesting question though is how the subject moves her hand before the target is found. We addressed human observers' hand movement strategies from the following four aspects.

#### 1) Did human observers move before the target was found?

**Figure 7:**
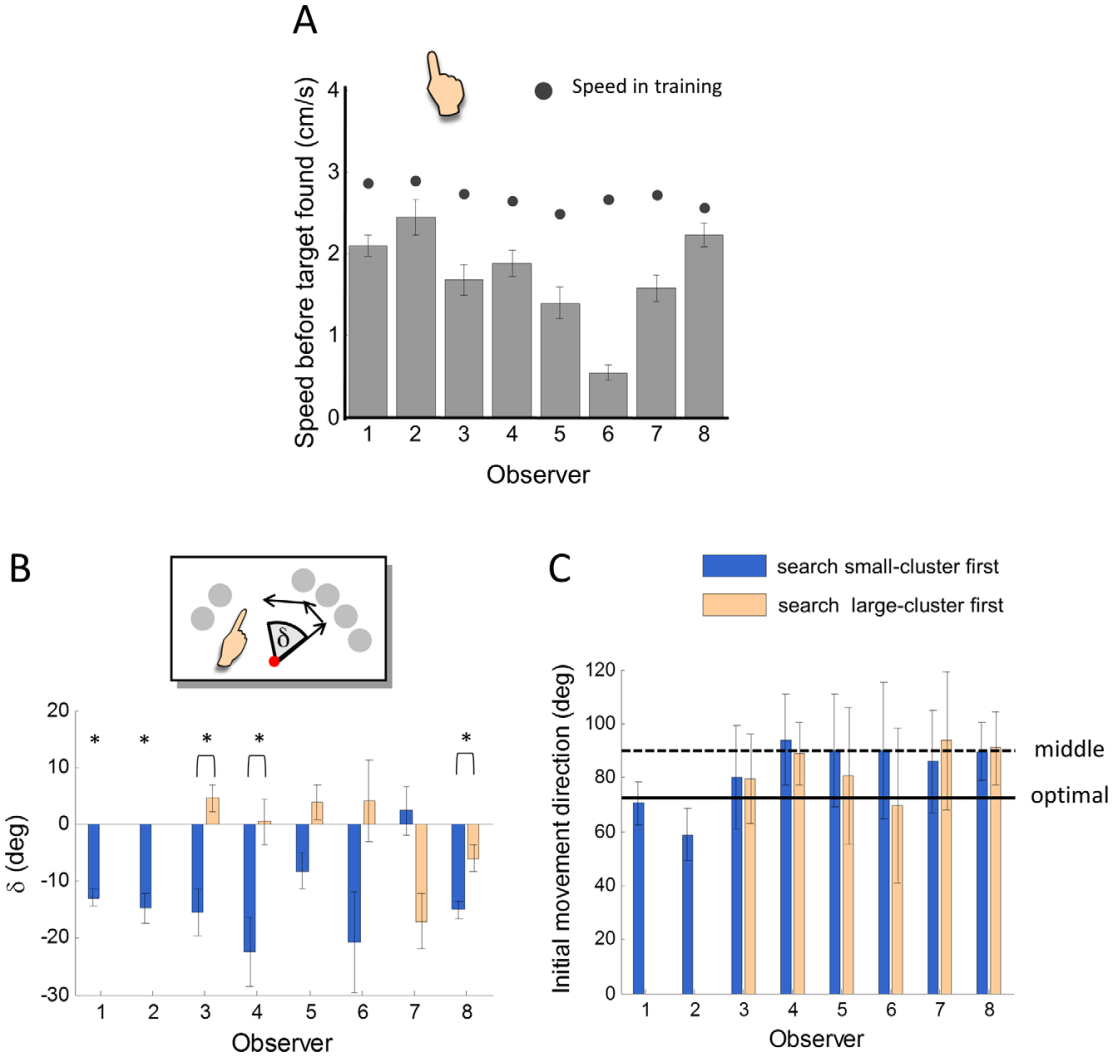
Hand movement strategies in the search-reach task. **A. Mean movement speed before the target was found**. Error bar denotes the 95% confidence interval. Before the target was found, if the observer did not move at all, it might cost her more than 30% of the rewards. Observers' actual movement speed was significantly larger than zero, but six of the eight observers moved significantly and much slower than they did in the training of reach. **B. The update of movement directions for different orders of visual search**. In a trial where the observer searched no fewer than three objects before locating the target, we compared the position of the finger at the end of fixating the third object with that of the first object. The difference of angle (inset), 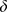, is plot separately for each observer and the search strategies of small-cluster first and large-cluster first. Positive for shifts towards the small cluster, negative for shifts towards the large cluster. Error bar denotes the standard error. The large-cluster first strategy for O1 and O2 had fewer than five valid trials and were not plot therefore. By the optimal hand movement strategy, the observer should shift towards the large cluster when searching the small cluster first, thus a negative 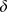, and vice versa. Six of the observers (O1∼O6) showed such a tendency. When searching small cluster first, five observers shifted significantly more towards the large cluster than that of large-cluster first, or significantly towards the large cluster (for observers who had not a large-cluster first comparison). Stars denote significant difference. **C. Mean initial movement direction for different orders of visual search**. For each trial with no less than one object searched before the target, the initial movement direction was defined as the direction from the starting position to the position of the finger at the end of fixating the first object. The direction was quantified in a polar coordinate centered at the starting position. The direction to the right was 0 degree; to the up, 90 degrees; to the left, 180 degrees. Error bar denotes the 95% confidence interval. The solid line denotes the optimal initial movement direction, which is towards the centroid of the six objects. The dash line denotes the middle of the two clusters. There was no significant difference in initial movement direction between the search orders. According to the mean initial movement direction, only two of the eight observers did not deviate significantly from optimal, while six of them were indistinguishable from a possible strategy of moving towards the middle of the two clusters.

Yes, but six of the eight observers moved markedly slower than they had did in the training of reach phase (Figure 7A). In each trial of the search-reach task, we could separate the visual search into a series of fixations and saccades. Before the observer saccades to the target (except when the target is at the last position searched), we are sure she does not know the position of the target. The speed shown in Figure 7A was computed for the period from the onset of the trial to the time the observer started a saccade to the target. According to a two-tailed one-sample Student's *t*-test for each observer, the differences between this speed and the average movement speed during the training were significant for six out of eight observers and in the same direction for the remaining two.

In the search-reach task, for a typical observer (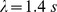 and 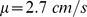), if the observer does not move before the target is found, she would be expected to receive only 61%∼69% of the maximal gain predicted by our model of optimal eye-hand coordination. Obviously the human observers benefited from moving before the target was found, but they did not make the most of this possibility by moving at their full speed. An observer commented after the experiment that he moved slowly because he felt it was not wise to move too fast when he was uncertain of the location of the target.

Is it possible that some observers might have benefited from moving slowly or even not moving before the target was found? We considered one possibility. Suppose that, due to visual and motor variability, the actual movement of the hand may deviate from the planned direction, particularly if the eye is engaged elsewhere. If the hand is moving in a wrong direction, moving at full speed would amplify the effect of any error. Suppose the angular error of hand movement was a Gaussian distribution of a standard deviation of 5 deg, a pessimistic estimate given previous estimates of human pointing performance without visual feedback [26]. For each observer, we simulated movement trajectories but now varying speed from zero to the observer's full speed while adding angular errors to the portion of the hand trajectory before the target was identified. We assumed that the observer might search the small cluster first (123456) or the large cluster first (654321) and simulated for these two orders separately.

In the simulation, all the observers' maximum expected gain increased monotonically with speed (Figure S5), regardless of their increased motor error and search order. That is, moving slower than their full speed would reduce their monetary rewards.

#### 2) Did human observers accommodate their hand movement to their search?

Each time an object was identified as a distractor, the object was excluded from the possible targets. As we discussed earlier under the model of optimal eye-hand coordination, given a specific order of search, the optimal hand movement strategy could be well approximated by the strategy of aiming at the centroid of the possible targets. Therefore, if human observers accommodate their hand movement to their search, we expected them to update their movement direction towards the centroid of the as yet unsearched objects. That is, in a trial, if the observer searched the small cluster first, she should shift towards the large cluster when the objects in the small cluster have been identified as distractors, and vice versa.

For trials which had no fewer than three objects fixated before the target is found, we evaluated the change of movement direction by computing the difference between the position of the finger at the end of fixating the third object and that of the first target. We used the angle 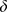 (inset of Figure 7B) to describe the difference. Except for Observers O1 and O2 who almost always searched the small-cluster first, we plotted the mean 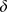 across trials separately for the visual search strategies of small-cluster first and large-cluster first for each observer (Figure 7B). Seven of the eight observers had the appropriate tendency to move towards the large cluster (negative 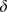) when searching the small cluster first. Four of the six observers (excluding O1 and O2) correctly moved towards the small cluster (positive 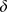) when searching the large cluster first. We tested whether there was a significant shift of movement in the correct direction (for O1 and O2) or whether there the shift of movement differed for the two search strategies in the correct direction (for the rest observers). According to a two-tailed one-sample Student's *t*-test (for O1 and O2) or a two-tailed two-sample Student's *t*-test (for the rest observers), five of the differences were significant (marked with star in Figure 7B).

To summarize, five of the eight observers appropriately adjusted their hand movement based on current information from the visual search strategy they were using.

#### 3) Did human observers move towards the optimal aim point?

Although most of the observers correctly updated their movement direction with the progressing visual search, only a few of them moved in the direction that agreed with the optimal model of hand movement.

For trials with no less than one object fixated before the target, we defined the direction of initial movement as the direction from the starting position to the position of the finger at the end of fixating the first object. We characterized it in the angle in a polar coordinate system that centered at the starting position and ran counter-clockwise from the direction to the right. The mean initial movement direction across trials is shown in Figure 7C for each observer. According to a two-tailed one-sample Student's *t*-test for each observer, only two of the eight observers (O1 and O6) did not deviate significantly from optimal, while six of them (including O6) were indistinguishable from a possible strategy of moving towards the middle of the two clusters.

#### 4) Did the hand simply follow the eye?

In Figure 7B, you may notice that, for most observers, when the eye shifted from the small cluster towards the large cluster, the hand shifted towards the large cluster as well, and vice versa. This agreed with the “hand-follows-eye” strategy mentioned in the Introduction. However, we had at least two reasons to reject the hand-follows-eye strategy as an explanation of the observers' performances. First, the initial movement direction was little influenced by the order of visual search (Figure 7C), inconsistent with what would be predicted by the hand-follows-eye strategy. Second, if the hand followed the eye, when the fixation was switched from one object to another, the angle of shift in hand movement direction should equal to the angles between objects. The centers of two adjacent objects in the same cluster were separated by 15 deg. The adjacent two objects in the two clusters were separated by 60 deg. If the observer started search from the small cluster, when examining the third object, the fixation was at least 60 deg away from the initial fixation. For searches starting from the large cluster, the third fixation should be at least 30 deg away from the initial fixation. However, the corresponding direction shifts in hand movement were far smaller (Figure 7B).

## Discussion

We tested the optimality of human strategies of eye-hand coordination in a task that involved finding and then touching a target among distractors as rapidly as possible. To minimize the overall time of visual search and hand movement and thereby to maximize expected gain, the observer needed to search the possible locations of the target in a specific order and alter her hand movement repeatedly in response to new visual information.

In such a task, the optimal strategies of visual search and hand movement were inter-related and jointly determined by the time required to identify an object and move the hand to touch it. For objects divided into two uneven clusters, the optimal visual search strategy was to search the small cluster first and then the large cluster. The optimal hand movement strategy was to move towards the centroid of the objects that had not been searched yet.

We examined human observers' hand movement and visual search strategies separately. We found that observers did (correctly) move before the target was found and most observers updated their movement direction correctly contingent on the progress of their search.

This outcome is consistent with previous studies that show motor compensation for increased visual and motor uncertainty [27]–[29], and particularly, with those that show people vary their trajectories of reach based on the spatial distribution of possible target locations [30], [31].

Sensitivity to probabilistic structures does not guarantee the optimality of movement under uncertainty. In our task, where the optimal strategy of hand movement could be clearly defined, we found that human observers significantly departed from optimal: Before the target was found, they did not move in their full speed and, at the beginning of their movements, only two out of eight correctly move in the optimal direction.

As to the visual search strategy, most of the observers failed to prefer the optimal visual search strategy. They started their search from the small cluster and the large cluster equally often. This failure is probably due to the indirect link between eye movements and the ultimate rewards. In the search-reach task, the better search orders do not help to shorten the search time itself but instead serves to shorten the movement time of the hand to the target. Although we investigators could model these indirect benefits or costs of eye movements [32], it is an open question whether human observers could.

The sub-optimality of visual search or hand movement strategy might have been a result of inability to plan eye movements and hand movements independently. For instance, the endpoints of the eye and the hand are correlated in rapid reaching [33]. However, we considered this possibility to be improbable. The observers did not tend to saccade to and move toward the same position. Nor can the sub-optimality be attributed to the constraints of the visual system. The usage of both the small-cluster first and large-cluster first search strategies is evidence that observers had the ability to use either. They simply did not realize one was a better strategy than the other.

Performing visual search and hand movement at the same time might lead to reduced performance in one or even both tasks. The observer might need a longer time to examine an object or the observer might have a larger variance in hand movement speed and thus have to slow down in order not to violate the time limit. However, as shown in Figure S1 and Figure S2, for most observers, the search slope was not larger for the test task and neither did the variance in hand movement speed. The slowing down of hand movement in the test task was more likely due to a choice of strategy, rather than constraints on motor control.

To conclude, people intelligently coordinate their hand with their eye in an uncertain environment. However, most of them did not use the eye and/or hand strategies that would have maximized the expected gain of the overall activity. Our study opens up the question: To what extent can the cost of one effector (e.g. hand) be taken into account in the movement planning of another effector (e.g. eye) of the same organism?

There are evidently costs of control in eye movements alone and in hand movements alone but these costs are consistent with near-optimal performance in the many tasks reported in the literature and reviewed in the introduction. In our task subjects must plan movements of two effectors (eye and hand) and it is very plausible that the sub-optimality we observe is due to a cost associated with planning two inter-related tasks (a “cost of coordination”). Testing this conjecture is a worthwhile direction for future research.

## Supporting Information

Figure S1 **Search slope: free search vs. search-reach.**
(PDF)

Figure S2 **Movement speed: reach training vs. search-reach.**
(PDF)

Figure S3 **Histogram of the movement time after the target was found (post-found time).**
(PDF)

Figure S4 **Percentage of specific search orders used by each observer.**
(PDF)

Figure S5 **Maximum expected gain as a function of hand movement speed before target was found.**
(PDF)

Video S1 **Eye-hand dynamics on one trial.**
(AVI)
