# Supplementary material for: Very Slow Search and Reach: Failure to Maximize Expected Gain in an Eye-Hand Coordination Task
Source: PLoS Comput Biol. 2012 Oct 11;8(10):e1002718. doi: 10.1371/journal.pcbi.1002718 (PMC3469464; doi:10.1371/journal.pcbi.1002718)
Supplement: Figure S1 — Search slope: free search vs. search-reach. (PDF) [file pcbi.1002718.s001.pdf]

Did visual search become slower when hand movement was involved?

In the search-reach task, observers moved their finger when they were searching for the target. Did the hand movement interfere with the visual search?

We compared each observer's visual search performance in the search-reach task to that in the training of free search task. Figure S1 shows that the search time, as a linear function of number of objects searched was little influenced by the tasks.

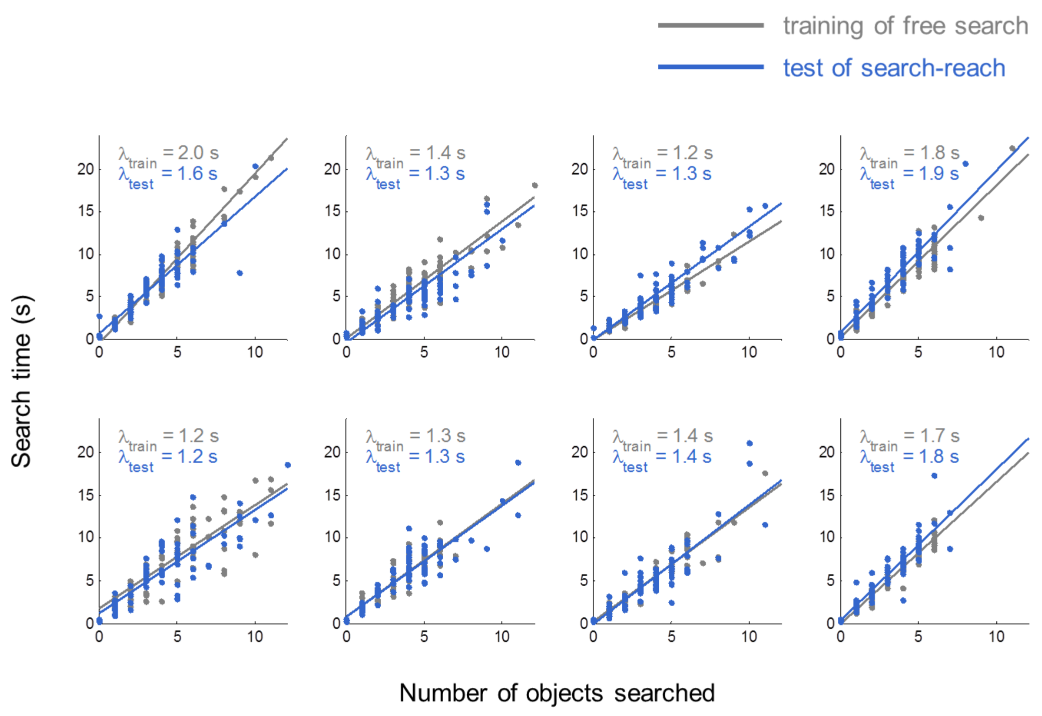

Figure S1.

Search slope: free search vs. search-reach.

Search time is plotted as a linear function of number of objects searched. Each panel is for one observer. Black dots and line denote data and linear fit for the training of free search task. Blue dots and line denote data and linear fit for the search-reach tasks. λ denotes the slope of the fitted line, indicating the search time per object.
