# Supplementary material for: Very Slow Search and Reach: Failure to Maximize Expected Gain in an Eye-Hand Coordination Task
Source: PLoS Comput Biol. 2012 Oct 11;8(10):e1002718. doi: 10.1371/journal.pcbi.1002718 (PMC3469464; doi:10.1371/journal.pcbi.1002718)
Supplement: Figure S2 — Movement speed: reach training vs. search-reach. (PDF) [file pcbi.1002718.s002.pdf]

Why didn't the observers move at the maximum speed of 4 cm/s?

Due to the neural variability in movement preparation (Churchland et al., 2006) or the neuromuscular noise in movement execution (de C. Hamilton et al., 2004), the actual instantaneous speed of observers' hand movement would deviate from the planned speed from time to time. That is, if the observer planned a speed of 4 cm/s, she would have a good chance to exceed the speed limit and thus fail the task. In Figure S2, we plotted a histogram of the instantaneous speed averaged across time windows of 0.2 second for each observer, in the training of reach and in the test of search-reach before the target being found. With the variance of movement speed shown in the figure, it seems reasonable to plan a speed of 2.5-2.9 cm/s, which would be the actual mean speed. If, for example, a subject planned a mean speed of 4 cm/s, they would have a 68% probability of exceeding the speed limit; while with a planned speed of 2.9 cm/s this probability would only be 0.4%.

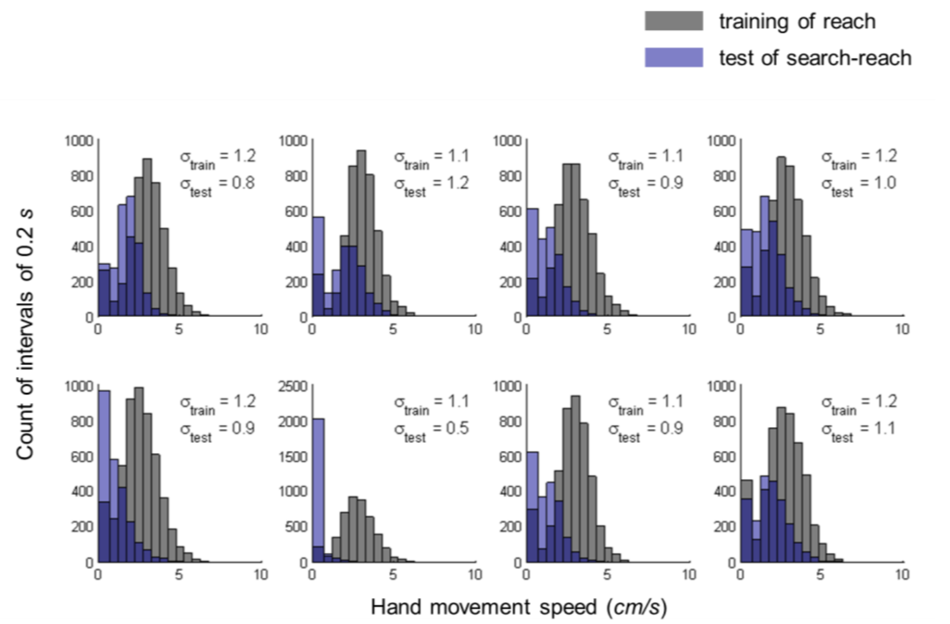

Figure S2. Movement speed: reach training vs. search-reach. The histogram of instantaneous hand movement speed (averaged across intervals of 0.2 *s*) was plotted for each observer. Each panel is for one observer. Gray and purple respectively denote the training of reach and the search-reach tasks. Dark gray denotes their overlap. For the search-reach task, the concerned hand movement speed was that before the target was found. σ denotes the standard deviation.

Did the slow hand movement in search-reach reflect a difficulty in motor control?

In the search-reach task, before the target was found, observers moved their finger much slower than they did in the training of reach task. Moving slower would reduce their expected gain. Why did observers move slower in the search-reach task? One possibility is, before the target was found, observers had to move their hand and searched for the target at the same time. As a result of a higher load of motor control than the single task of reaching, the observer might have a larger variance in hand movement speed and thus have to slow down in order not to violate the time limit. Figure S2 is inconsistent with this possibility: For most observers, the variance in hand movement was not larger in the search-reach task than in the training of reach task. The slowing down of hand movement in the test task was more likely due to a choice of strategy, rather than constraints on motor control.

Churchland MM, Afshar A, Shenoy KV (2006) A central source of movement variability. Neuron 52: 1085-1096.
de C Hamilton AF, Jones KE, Wolpert DM (2004) The scaling of motor noise with muscle strength and motor unit number in humans. Exp Brain Res 157: 417-430.
