# Supplementary figures and images for: Very Slow Search and Reach: Failure to Maximize Expected Gain in an Eye-Hand Coordination Task
Source: PLoS Comput Biol. 2012 Oct 11;8(10):e1002718. doi: 10.1371/journal.pcbi.1002718 (PMC3469464; doi:10.1371/journal.pcbi.1002718)

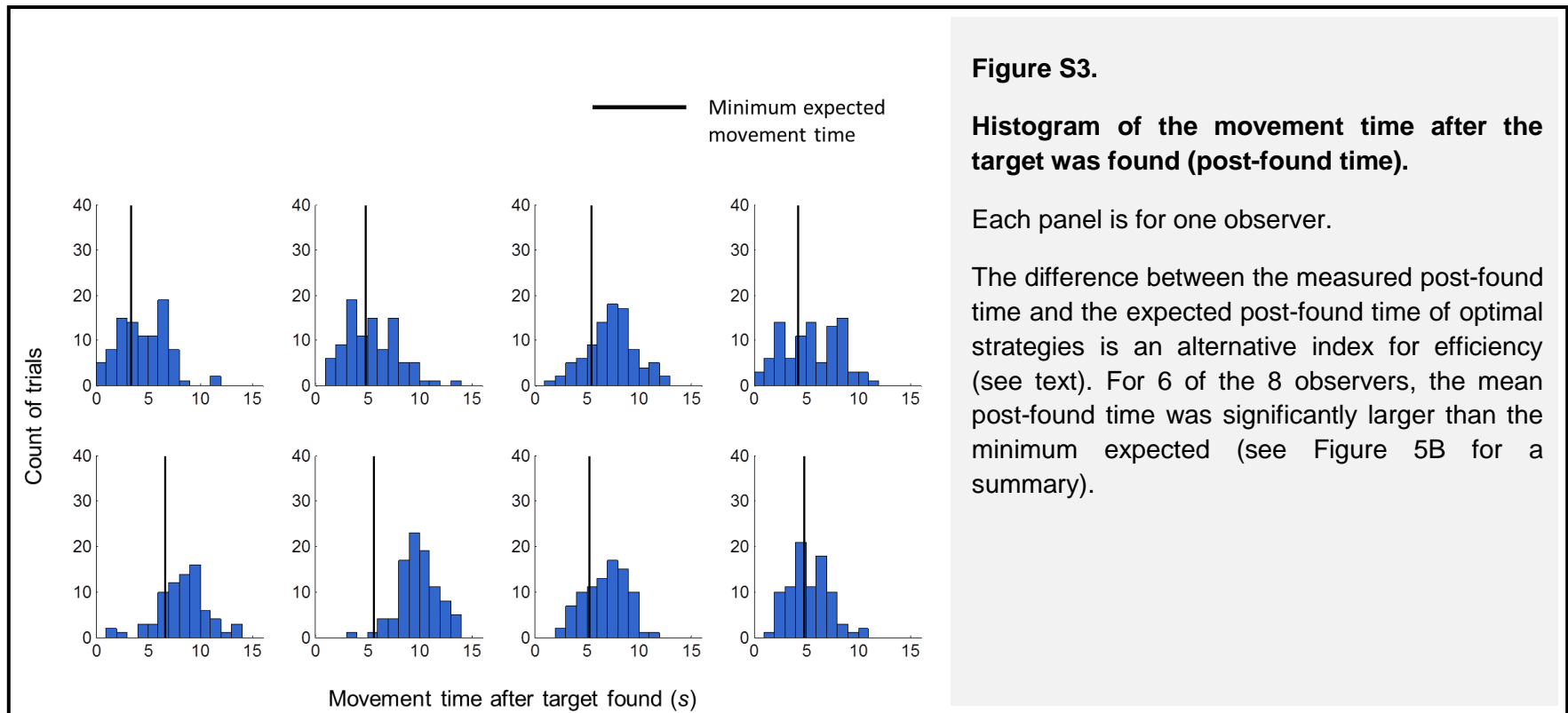

Supplement: Figure S3 — Histogram of the movement time after the target was found (post-found time). (PDF) [file pcbi.1002718.s003.pdf]

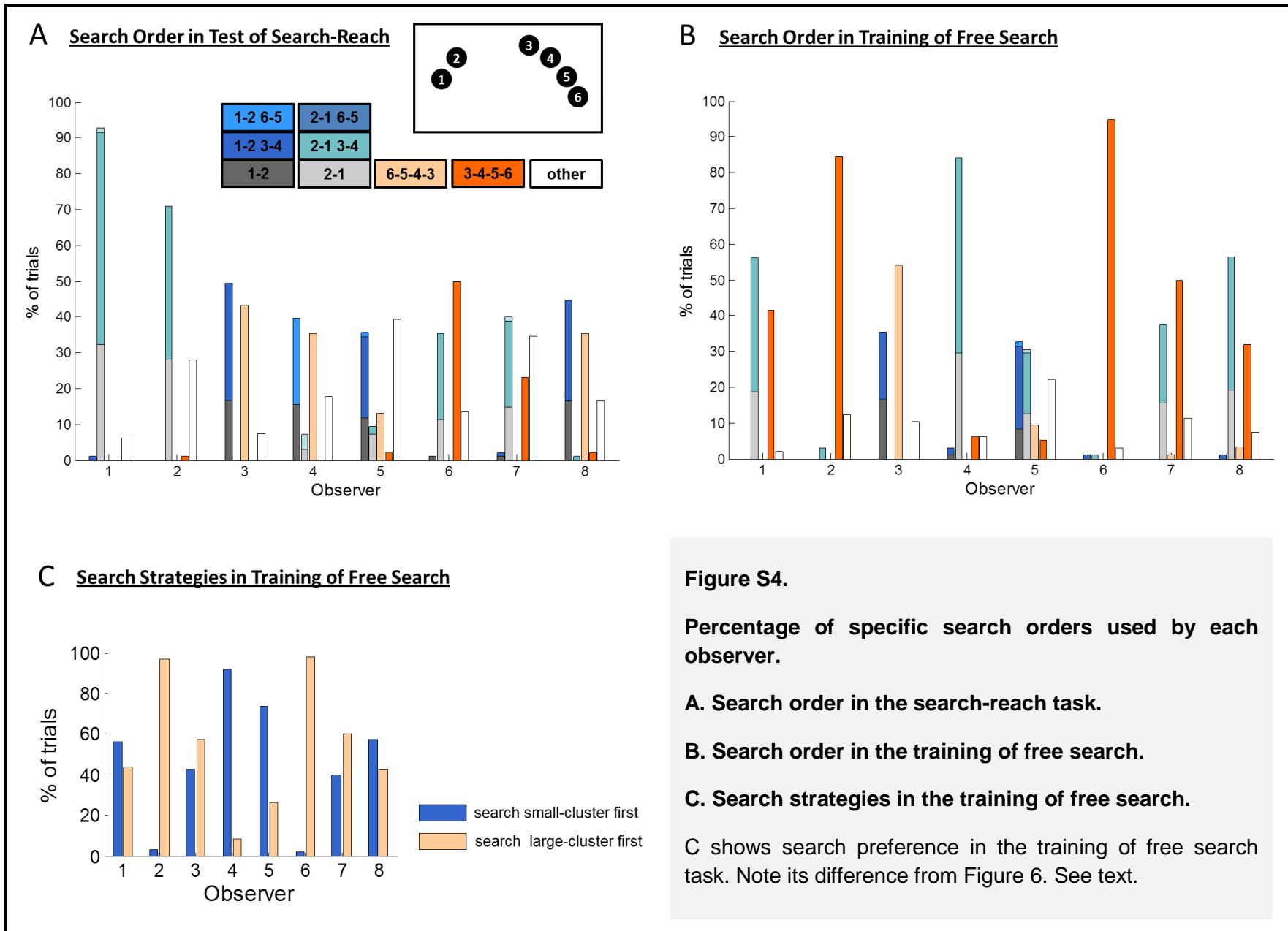

Supplement: Figure S4 — Percentage of specific search orders used by each observer. (PDF) [file pcbi.1002718.s004.pdf]
