# Supplementary material for: Very Slow Search and Reach: Failure to Maximize Expected Gain in an Eye-Hand Coordination Task
Source: PLoS Comput Biol. 2012 Oct 11;8(10):e1002718. doi: 10.1371/journal.pcbi.1002718 (PMC3469464; doi:10.1371/journal.pcbi.1002718)
Supplement: Figure S5 — Maximum expected gain as a function of hand movement speed before target was found. (PDF) [file pcbi.1002718.s005.pdf]

Could the observer benefit from moving slower before the target was found?

In the search-reach task, before the target was found, observers moved their finger much slower than they did in the training of reach task. Moving slower than their full speed would reduce their expected gain. Some observers stated after the experiment that they believed that they should not move fast when the target had not been found. It is possible that if moving towards a possible wrong direction at full speed would be costly. We tested the possibility that moving slower than the full speed could end up with a larger expected gain, assuming that the observer might randomly deviate from the planned direction of movement.

The results of our simulation were not consistent with this possibility. As Figure S5 shows, all the observers' maximum expected gain increased monotonically with the movement speed, regardless of their motor error and search order.

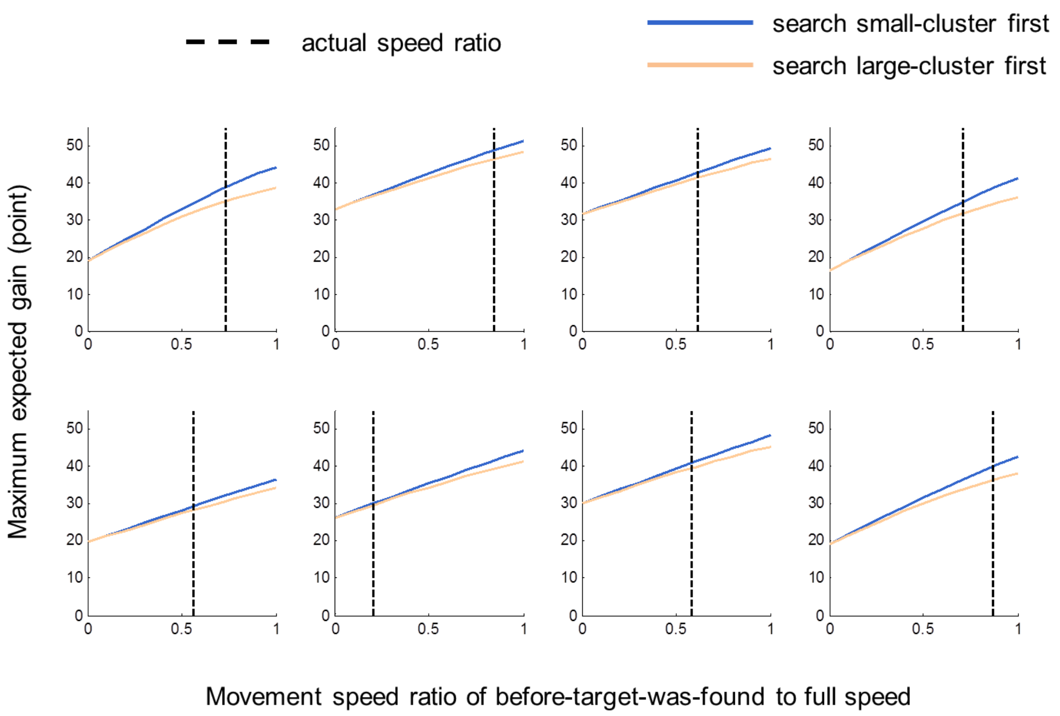

Figure S5.

Maximum expected gain as a function of hand movement speed before target was found.

Each panel is for one observer. The angular error of hand movement was assumed to be a Gaussian distribution of a standard deviation of 5 deg. Blue and orange lines denote the results for different assumed search orders. The dash line denotes the observer's actual speed (the full speed was normalized to be 1).

All the observers' maximum expected gain increased monotonically with the movement speed.
